# Pediatric Hand Surgery Training in Nicaragua: A Sustainable Model of Surgical Education in a Resource-Poor Environment

**DOI:** 10.3389/fpubh.2017.00075

**Published:** 2017-04-11

**Authors:** Mary Claire B. Manske, Jairo J. Rios Roque, Gabriel Ramos Zelaya, Michelle A. James

**Affiliations:** ^1^Shriners Hospital for Children Northern California, Sacramento, CA, USA; ^2^Hospital Infantil Manuel de Jesus Rivera “la Mascota”, Managua, Nicaragua

**Keywords:** pediatric hand surgery, orthopedic surgery training, Nicaragua, resource-poor environment, surgical education

## Abstract

Recent reports have demonstrated that nearly two-thirds of the world’s population do not have access to adequate surgical care, a burden that is borne disproportionately by residents of resource-poor countries. Although the reasons for limited access to surgical care are complex and multi-factorial, among the most substantial barriers is the lack of trained surgical providers. This is particularly true in surgical subspecialties that focus on life-improving, rather than life-saving, treatments, such as pediatric hand and upper extremity surgery, which manages such conditions as congenital malformations, trauma and post-traumatic deformities including burns, and neuromuscular conditions (brachial plexus birth palsy, spinal cord injury, and cerebral palsy). Many models of providing surgical care in resource-limited environments have been described and implemented, but few result in sustainable models of health-care delivery. We present our experience developing a pediatric hand and upper extremity surgery training program in Nicaragua, a resource-limited nation, that grew out of a collaboration of American and Nicaraguan orthopedic surgeons. We compare this experience to that of surgeons undergoing subspecialty training in pediatric upper limb surgery in the US, highlighting the similarities and differences of these training programs. Finally, we assess the results of this training program and identify areas for further growth and development.

## Introduction

Although the origin of the adage “Give a man a fish, and you feed him for a day. Teach a man to fish, and you feed him for a lifetime,” is debated, the principle it conveys, that the acquisition of skills promotes self-sufficiency, can be found in numerous diverse philosophies. The ubiquity of this concept suggests that it is applicable to the acquisition of any scarce resource, including medical and surgical care.

## Background and Rationale

In 2015, the *Lancet* Commission on Global Surgery estimates that nearly 5 billion people, approximately two-thirds of the world population, do not have access to adequate surgical care (1). Not surprisingly, this burden is borne disproportionately by people in resource-poor nations (1), with the poorest third of the world population receiving only 3.5% of all surgical procedures (2). Given that an estimated 30% of global diseases are treatable with surgical intervention, the lack of access to safe, timely, and appropriate surgical care results in substantial morbidity, mortality, and disability. Additionally, although the relationship between health and socioeconomic status is complex, several studies have established an association between poor health (especially in childhood) and poor socioeconomic status in both developed and developing nations (3, 4). Consequently, in addition to a moral imperative to address disparities in access to adequate surgical care, the dedication of resources to improve surgical care in resource-poor environments will likely have economic benefits at both the individual and societal level, the ramifications of which are likely to be far reaching.

The reasons for inadequate surgical care in resource-poor environments are multi-factorial, including inadequate infrastructure (facilities, electricity, water), limited physical resources (surgical and anesthesia supplies and equipment), lack of adequately trained anesthesia and surgical providers, and under-utilization of surgical services for financial, cultural, and religious reasons (5, 6). Moreover, strategies to improve global health often focus on infectious and communicable diseases, rather that surgically treated diseases, which are often characterized as expensive and technologically demanding to treat, despite emerging evidence that suggest that surgical care is cost effective (7). Although there are many reasons for lack of surgical care, the shortage of trained surgical providers is among the most significant barriers to essential surgical care worldwide (8).

If we accept the wisdom of the “teach a man to fish” philosophy, part of the solution to limited access to surgical care may be to increase the number of qualified surgeons by providing training experiences in surgical disciplines in which there are currently inadequate numbers of trained local providers. The goal of such a training program would be to develop surgeons who are not only capable to delivering surgical care but who are also able to mentor and train additional surgeons (residents, colleagues) to create a sustainable supply of knowledgeable, competent surgical providers. We describe our experience developing collaboration between American and Nicaraguan surgeons to provide pediatric hand and upper extremity surgery in Nicaragua.

## Competencies and Standards

In the US, hand surgery fellowship training is available to graduates of ACGME-accredited residencies in orthopedic surgery, plastic surgery, and general surgery, and pediatric orthopedic training is available to orthopedic surgery residency graduates. Following hand surgery or pediatric orthopedic fellowship training, approximately five US surgeons each year elect to obtain an additional 6 months of training in pediatric hand surgery, to prepare them to care for children with congenital hand malformations, neuromuscular conditions such as brachial plexus birth palsy, spinal cord injury, cerebral palsy, and post-traumatic deformities. These highly specialized surgeons acquire the diagnostic skill set of pediatric orthopedists by gaining an understanding of the impact of growth on musculoskeletal deformities, the treatment of syndromes, and the technical skill set of hand surgeons (training in complex reconstructive procedures that treat pediatric hand conditions).

While no formal accreditation of pediatric hand surgery exists, this discipline, a hybrid subset of pediatric orthopedic surgery and hand surgery, has become well established in the US. Most children’s hospitals now include a pediatric hand surgeon on their staff and the Pediatric Hand Study Group (PHSG), established in 1995, meets twice-yearly, performs multicenter research projects, and presents an international award each year for the best pediatric hand article (9–14). The senior author (Michelle A. James) is a founding member of the PHSG and has directed a pediatric hand fellowship training program at Shriners Hospital for Children Northern California since 2009, training eight pediatric hand fellows (http://shrinerschildrens.org/pediatric-hand-upper-extremity-surgery-fellowship). In this training program, pediatric hand fellows typically perform approximately 100 cases in a 6-month period (Table 1).

**Table 1:** **Shriners Hospitals for Children Northern California pediatric hand fellow experience**.

Diagnosis (average number of cases per fellow)	Procedures performed
Brachial plexus birth palsy (12)	Brachial plexus exploration and reconstruction with sural nerve autograft or nerve transfers
	Open subscapularis/pectoralis major release; teres major/latissimus dorsi tendon transfer
	Arthroscopic anterior shoulder capsule and subscapularis release; teres major/latissimus dorsi tendon transfer
	Botox injection for shoulder internal rotation contracture
	Biceps re-routing for supination contracture
	Distal humerus external rotation osteotomy
	FCU to ECRB tendon transfer
Retained hardware (7)	Removal of deep implant
Syndactyly/symbrachydactyly (6)	Syndactyly release with/without full thickness skin graft
Trigger thumb (6)	Trigger thumb release
Elbow trauma/sequela of elbow trauma (6)	Lateral or medial condyle fracture non-union repair
	LUCL or MCL ligament reconstruction for elbow instability
	Elbow arthroscopy
	Corrective osteotomy of distal humerus malunion
	Elbow contracture release
	Elbow arthrotomy, excision of loose bodies, interposition arthroplasty
	LUCL reconstruction secondary to traumatic elbow instability
	ORIF lateral or medial condyle fracture
	Ulnar nerve decompression, transposition
	Closed reduction, percutaneous pinning supracondylar humerus fracture
	Distal humerus epiphysiodesis
Hand trauma/sequela of hand trauma (6)	ORIF scaphoid fracture or non-union
	Extensor tendon repair, central slip repair
	Flexor tendon repair or reconstruction, pulley reconstruction
	Centralization of extensor tendons for tendon subluxation
	Revision amputation of finger
	Interphalangeal joint fusion
	Phalanx osteotomy
	Digital nerve repair
	ORIF or percutaneous pinning of phalanx or metacarpal fracture
Sequela of forearm trauma (5)	Corrective osteotomy of radius and/or ulna
	Skin grafting for fasciotomies for compartment syndrome
	Forearm fasciotomy for compartment syndrome
Radial polydactyly (4)	Polydactyly reconstruction
Ganglion cyst (4)	Ganglion cyst excision
Hypoplastic thumb (4)	Thumb reconstruction
	Pollicization
	Epiphysiodesis for pollicization overgrowth
Burn injury (3)	Wrist/finger contracture release
	Burn excision and skin grafting
	PIP arthrodesis
Arthrogryposis (4)	First web space deepening
	Posterior elbow capsulotomy, triceps lengthening
	Index proximal phalanx rotational osteotomy
	Fractional lengthening flexor tendons
	Thumb MCP arthrodesis
Osteochondroma/multiple hereditary exostosis (3)	Osteochondroma excision
	Forearm osteotomy
	Radius hemiepiphysiodesis
Ulnar polydactyly (3)	Polydactyly reconstruction
Constriction band syndrome (3)	Constriction band release
	Revision amputation of finger for bony overgrowth
	Acrosyndactyly release
	First web space deepening
Cerebral palsy (3)	Lateral band re-routing
	Thumb MCP arthrodesis
	Wrist arthrodesis
	Pronator release
	Adductor pollicis, thenar release
	FCU to ECRB tendon transfer
Radial longitudinal deficiency/TAR (2)	Wrist centralization (bony or soft tissue)
Aperts syndrome (1)	Complex syndactyly release with full thickness skin graft
Osteochondritis dissecans lesion of capitellum (1)	OATS procedure
Hand/finger bone or soft tissue mass other than ganglion (2)	Excisional biopsy, curette, ORIF
Brachymetacarpia (1)	Metacarpal lengthening
Retained foreign body (1)	Removal of foreign body
Tetraplegia (1)	Forearm rotational osteotomy
	Tendon transfer
Soft tissue abscess (1)	Irrigation, debridement
Clasp thumbs (1)	Index dorsal rotation flap
Flexor tenosynovitis (1)	Irrigation debridement flexor sheath
Shoulder instability (1)	Capsular shift and stabilization
TFCC injury (1)	wrist arthroscopy and TFCC repair
Transverse deficiency (1)	Stump amputation for overgrowth
Clinodactyly (1)	Excision of bracketed epiphysis
Osteogenesis imperfecta (1)	Humerus, radius, ulna osteotomies
Cleft hand (1)	Cleft reconstruction
Trigger finger (1)	Trigger finger release, synovectomy, FDP slip excision
Carpal tunnel syndrome (1)	Carpal tunnel release
Camptodactyly (1)	PIP contracture release
Shaken baby, L hemiplegia (1)	Fractional lengthening flexor tendons
Radioulnar synostosis (1)	Forearm osteotomy

## Learning Environment

### Nicaragua and the Nicaraguan Health System

Nicaragua is the largest country in Central America by land mass area, approximately the size of the stat of New York in the US, and has a population of 6.1 million people. The majority of the population lives in the Pacific region of the country, along the western coast, with 25% of the total population in the capital city Managua (15). Nicaragua also is the poorest country in Central American and the second poorest country in the Western Hemisphere (16) with nearly 50% of the population living under the poverty line and a per capita gross national income of US $2,720 (17).

The Nicaraguan health-care system has three tiers with both private and public components. The private health-care sector serves Nicaraguans with the financial resources to pay for care, approximately 10% of the population. The public sector includes the Nicaraguan Social Security Institute (Instituto Nicaraguense de Seguridad Social or INSS), which covers salaried government workers (10–20% of the population) and the Ministry of Health (Ministerio de Salud or MINSA), which provides health-care services for the remainder (approximately 70–80% of the population). In addition to being the predominant health-care provider in Nicaragua, MINSA is also the health-care regulatory agency and is responsible for all matters related to the provision of healthcare, from establishing health-care policy and government priorities to the salary schedule for providers (16).

### Medical Education and Orthopedic Surgery Residency in Nicaragua

There are both public and private medical schools in Nicaragua. Admission to public medical school is based on the results of a standardized admissions test, and tuition is covered by the government. Medical education in public medical schools lasts 8 years after high school (5 years of medical studies, 1 intern year, 2 years of social service). By contrast, private medical schools (American University, Catholic University, Military School) charge tuition and last 6 years (2 years of social service are not required). Following medical school, physicians wishing to become orthopedic surgeons must be accepted into an orthopedic surgery residency. These are administered by either MINSA or the National Autonomous University of Nicaragua (Universidad Nacional Autónoma de Nicaragua or UNAN). Most programs last 4 years. In 2015, 55 residents (including 10 women) were enrolled in orthopedic surgery residency in Nicaragua (18). In contrast to the US, where nearly 90% of graduating orthopedic surgery residents matriculate into an orthopedic subspecialty fellowship (19), there are no subspecialty orthopedic fellowships available in Nicaragua, although some residents complete an additional year of residency focusing on a particular subspecialty, and a small number seek subspecialty training in other countries, but due to financial and regulatory barriers, few surgeons pursue fellowship training.

According to the Asociación Nicaragűense Orthopaedia y Traumatología (ANOT), there were 210 actively practicing orthopedic surgeons in Nicaragua, about half of whom practice in Managua [3.5 orthopedic surgeons for every 100,000 people, compared with 8.5 per 100,000 in the US (19)]. However, not all Nicaraguan orthopedic surgeons belong to ANOT, so this estimate may not be accurate. Few Nicaraguan orthopedic surgeons have subspecialty training in pediatric orthopedic surgery or hand surgery, and no surgeons are trained specifically in pediatric hand surgery, a unique discipline that includes some of the most intricate and complex surgery in orthopedics. Other than service trips of surgeons from developed countries, pediatric hand surgeons are not available to treat congenital hand malformations, neuromuscular disorders (spinal cord injury, cerebral palsy, brachial plexus palsy), and post-traumatic deformities.

### La Brigada de las Manos

In 2009, under the auspices of Health Volunteers Overseas (20), a relationship was forged between three of the authors (Jairo J. Rios Roque, Gabriel Ramos Zelaya, and Michelle A. James), along with two additional members of the orthopedic and traumatology physician staff of a pediatric hospital in Managua, Nicaragua (Hospital Fernando Velez Paiz). In 2014, Hospital Velez Paiz was damaged by an earthquake, and the orthopedic staff and the pediatric hand surgery program (La Brigada de las Manos) transferred their services to Hospital Infantil Manuel de Jesus Rivera (Hospital “La Mascota”), the largest public children’s hospital in Nicaragua. Both Hospital Velez Paiz and Hospital La Mascota are administered by MINSA.

**Figure 1:**
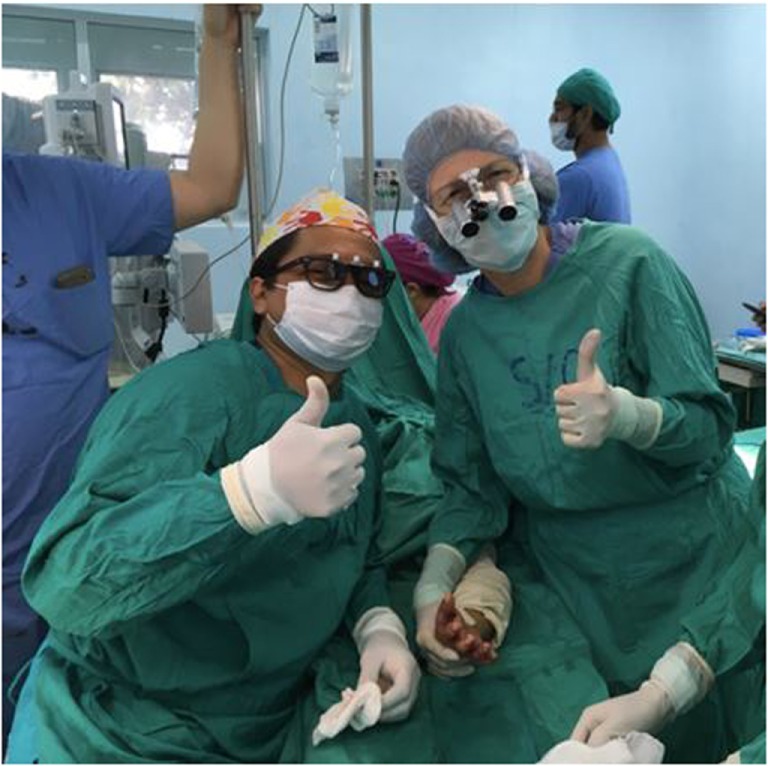
**Jairo J. Rios Roque, MD and Michelle A. James, MD following a syndactyly release procedure performed at Hospital “La Mascota” in Managua, Nicaragua**.

At the time of the first Brigada de las Manos trip in 2009, it was apparent that there existed in Nicaragua a large population of children with hand conditions, with little treatment available. The orthopedic surgeon sub-director of the children’s hospital (Gabriel Ramos Zelaya) requested that the Brigada provide hand surgery training to one of the junior orthopedic surgeons (Jairo J. Rios Roque), so that he could provide ongoing care between Brigada trips, and eventually gain expertise to care for complex pediatric hand problems independently. Jairo J. Rios Roque’s practice has been based at a public children’s hospital since 2009 and he has extensive experience, although no formal training, in pediatric orthopedic surgery, and a strong interest in hand surgery. The senior author (Michelle A. James) investigated various options for international hand fellowships in the US, and discovered that licensing and credentialing requirements virtually precluded hands-on training in the US for Jairo J. Rios Roque. Together, the sub-director and senior author developed a model of training that includes:

(1) Twice-yearly week-long Brigada visits. The Brigada includes two experienced pediatric hand surgeons (Michelle A. James and another member of the PHSG), a pediatric orthopedic occupational therapist, and additional volunteers. During these visits, the Brigada surgeons work with Jairo J. Rios Roque and the La Mascota staff to see approximately 100 children in clinic and to schedule and perform 20–25 operations (see Table 2). Jairo J. Rios Roque functions as a pediatric hand fellow, performing at least half of the cases together with a Brigada surgeon (Figure 1). The Brigada surgeons also train Nicaraguan residents. Jairo J. Rios Roque provides post-operative care for patients, communicating with Brigada surgeons by email as indicated. In addition, based on his performance in clinic and surgery, he is credentialed by the Brigada members to perform certain pediatric hand operations between trips.
(2) Nicaraguan surgeon visits to the US. As part of the planned training curriculum, Jairo J. Rios Roque visits the senior author every 1–2 years for 2–3 weeks (three visits between 2009 and 2016) where he observes clinic and surgery. He obtained a Pediatric Orthopaedic Society of North America international scholarship to attend a pediatric orthopedic meeting and visit two centers, in addition to attending the World Symposium on Congenital Hand Surgery in Dallas, TX, USA. At each Brigada visit, Jairo J. Rios Roque presents patients whom he has operated on since the previous visit to the visiting surgeons, who critique his results and provide feedback.

**Table 2:** **Operative cases performed by La Brigada de las Manos 2009–2016**.

Diagnosis (total number of operative cases)^a^	Procedures (number)
Arthrogryposis (38) (Figure 2)	First web space deepening/dorsal index finger rotational flap/thenar release (16)
	Finger contracture release/flexor tendon lengthening (6)
	Dorsal carpal wedge osteotomy (5)
	Posterior elbow release and triceps lengthening (5)
	Thumb MCP arthrodesis (3)
	Radius and ulnar osteotomy (2)
	ECU to ECRB tendon transfer (1)
Radial polydactyly (27) (Figure 3)	Polydactyly reconstruction
Syndactyly (25) (Figure 4)	Syndactyly release
Sequela of hand/finger trauma (16)	Repair of phalanx malunion/non-union (5)
	Tenolysis/tendon reconstruction (5)
	Revision finger amputation/ray resection (4)
	MCP capsule release, Z plasty, skin graft (1)
	Kutler advancement flaps (1)
Constriction band syndrome (12) (Figure 5)	Acrosyndactyly release with full thickness skin graft (8)
	Band excision (3)
	First web space deepening (1)
Sequela of forearm trauma (11)	Corrective forearm osteotomy for forearm malunion or missed Monteggia fracture (6)
	Ulnar shortening osteotomy for distal radius physeal arrest (2)
	Flexor tendon repair/reconstruction with median nerve reconstruction with sural nerve autograft (2)
	Distal radius/ulna epiphysiodesis (1)
Ulnar polydactyly (10)	Polydactyly reconstruction (10)
Sequela of elbow trauma (9)	Ulnar nerve transposition for tardy ulnar nerve palsy secondary to lateral condyle fracture (4)
	Ligament reconstruction for chronic elbow instability (3)
	Flexor tendon release/tendon transfer for Volkmann’s ischemic contracture (2)
Brachial plexus birth palsy (9)	Teres major/latissimus dorsi tendon transfer (3)
	Wrist arthrodesis (1)
	Biceps transfer (1)
	Radial derotational osteotomy (1)
	Pectoralis major and subscapularis release (2)
	FCU to ECRB tendon transfer (1)
Radial longitudinal deficiency/hypoplastic thumb (7) (Figure 6)	Pollicization (4)
	Thumb reconstruction (2)
	Wrist centralization (1)
Hand/finger mass (6)	Mass excision
Multiple hereditary exostosis (6)	Osteochondroma excision (5)
	Radius hemiepiphysiodesis (1)
Apert’s syndrome (6)	Syndactyly release (2)
	Thumb osteotomy (2)
	First web space deepening (1)
Trigger thumb (5)	Trigger thumb release
Sequela of burn injury (5)	DIP arthrodesis (2)
	Contracture release (3)
Madelung (5)	Dome osteotomy distal radius (3)
	Ulnar shortening osteotomy (2)
Cerebral palsy (5)	Tendon transfer (3)
	Wrist arthrodesis (2)
Poland syndrome/symbrachydactyly (3)	Syndactyly release
Nerve palsy (3)	PIP arthrodesis for finger contractures secondary to ulnar nerve palsy (1)
	Tendon transfers for radial nerve palsy (1)
	Carpal tunnel release (1)
Proximal radioulnar synostosis (3)	Corrective forearm osteotomy
Retained deep implant (2)	Removal of deep implant
Cleft hand (2)	Cleft closure
Juvenile arthritis (2)	Wrist arthrodesis
Macrodactyly (1)	Ray resection
Marfan’s syndrome (1)	Ligament reconstruction
Clinodactyly (1)	Osteotomy
Total number of cases (220)^a^	

*^a^Records of cases performed on two trips were lost, so this list represents 13 La Brigada trips*.

## Results to Date/Assessment

### Comparison to SHCNC Pediatric Hand Surgery Training Experience

**Figure 2:**
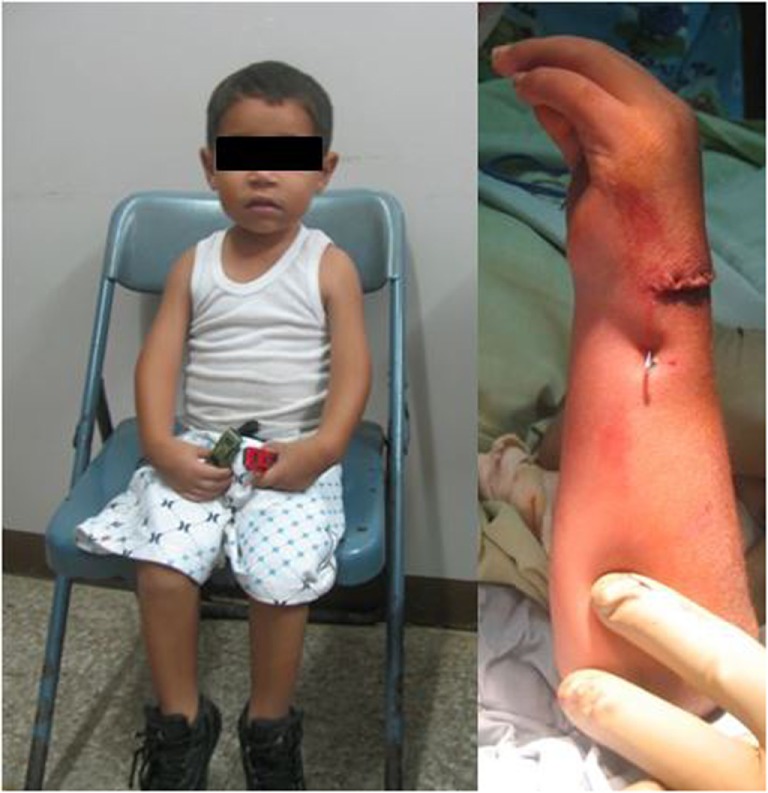
**A patient with arthrogryposis, limited elbow flexion, and wrist extension**. Note the flexed position of the wrists pre-operatively (image on the left). The patient was treated with a dorsal carpal wedge osteotomy to improve the position the wrist to neutral (image on the right).

**Figure 3:**
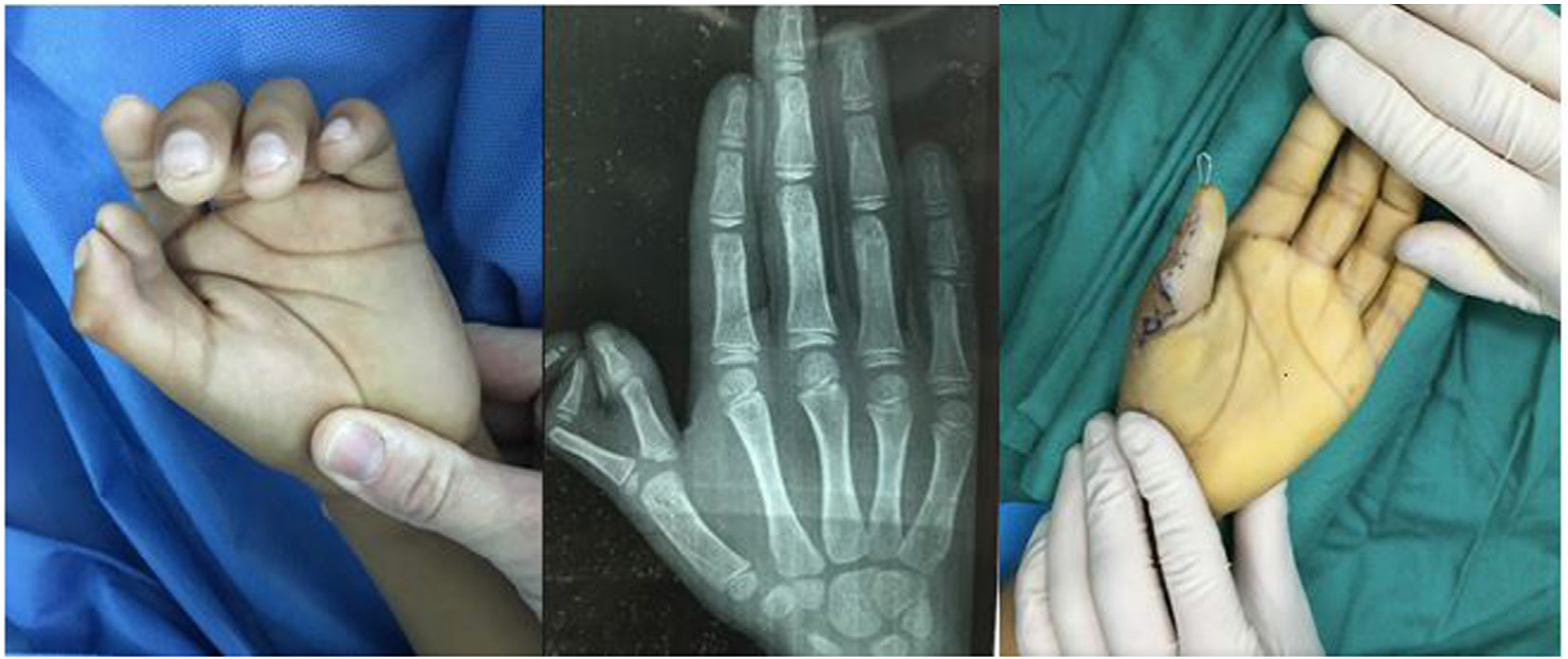
**Radial (thumb) polydactyly treated with reconstruction of the thumb to form a single stable thumb**.

**Figure 4:**
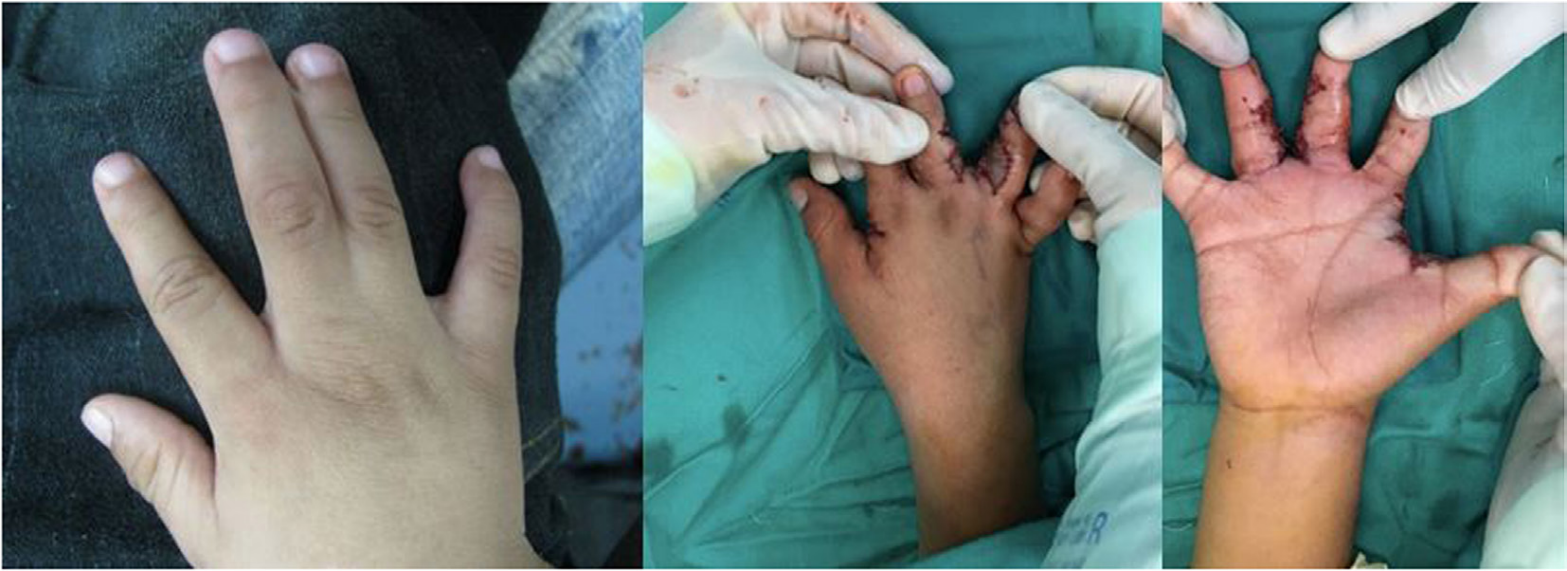
**Syndactyly (webbed digits) affecting the ring and long fingers treated with syndactyly release and skin grafting**.

**Figure 5:**
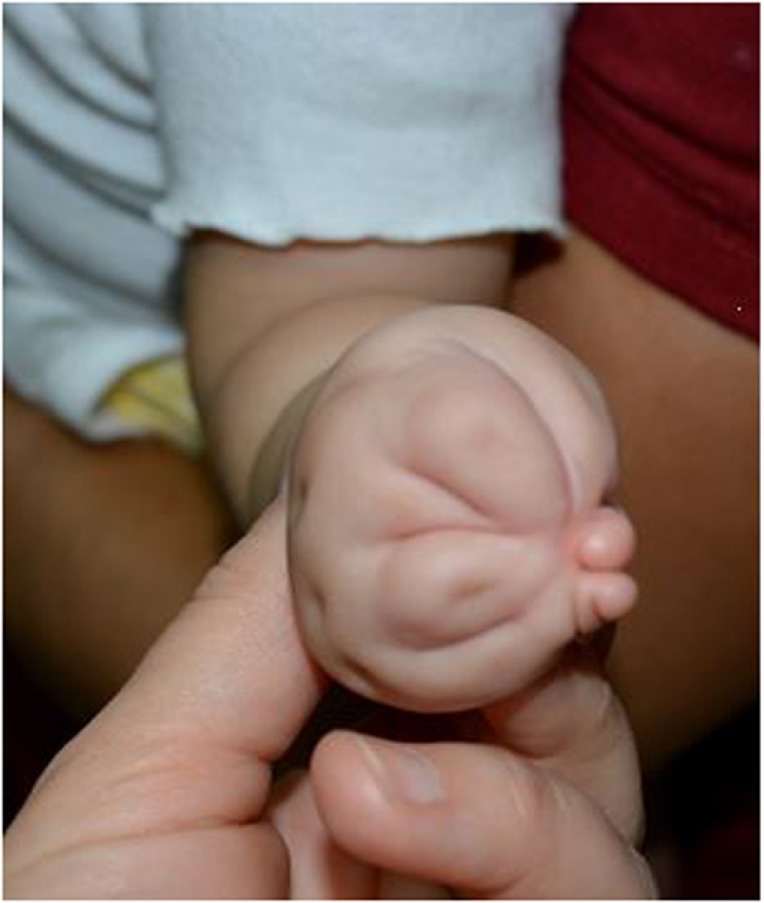
**Constriction band syndrome resulting in a complex syndactyly of the fingers**.

**Figure 6:**
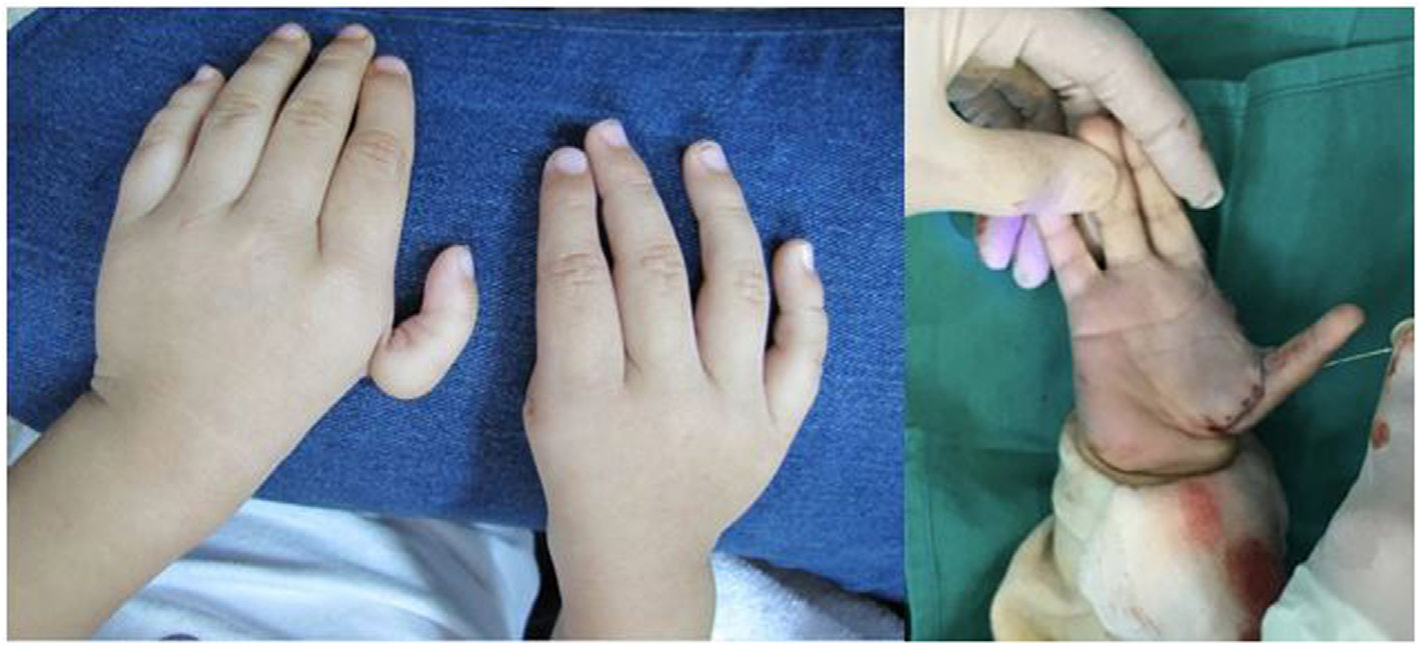
**Bilateral thumb hypoplasia with a severely underdeveloped thumb on the left hand and an absent thumb on the right hand**. This patient was treated with pollicization on both hands, in a staged fashion, which involves reconstructing and repositioning the index finger to function in the position of a thumb.

Although Jairo J. Rios Roque’s experience is not a consolidated period of six contiguous months, but is rather occurring over years in a series of 1 week intervals, it is similar in number of cases and types of cases to a typical pediatric hand surgery training fellowship in the US (see Tables 1 and 2). There is substantial overlap in the most frequent diagnoses and procedures, with surgeries for polydactyly, syndactyly, sequelae of upper extremity trauma, and neuromuscular conditions (arthrogryposis, cerebral palsy) being among the most common procedures performed (Figures 2–6). Moreover, the volume of operative cases performed by Jairo J. Rios Roque is comparable to those of the typical US pediatric hand surgery fellow during his or her 6-month fellowship. SHCNC pediatric hand surgery fellows typically perform 100 surgical cases during their 6-month training experience and, after 7 years, Jairo J. Rios Roque has performed approximately half of the 220 cases with the Brigada during that time, and observed many of the other cases. Finally, the Nicaraguan training experience employs the concepts of graduated responsibility, such that Jairo J. Rios Roque’s responsibility and ability to independently care for patients in clinic and the operating room increases, commensurate with his experience, skills and core competencies, benchmarks for determining Jairo J. Rios Roque’s progress, and ability to practice pediatric hand surgery independently.

Nevertheless, there are differences between the Nicaragua training experience and the SHCNC experience. The biggest difference is the lack of availability of hand fellowship training for Jairo J. Rios Roque as a prerequisite to pediatric hand surgeon training. Although hand fellowships focus on the care of adult hand problems, they provide training in the basic hand surgical skills needed to perform most pediatric hand surgical procedures. For this reason, Jairo J. Rios Roque’s training is prolonged, and will likely not ultimately include the most complex procedures, especially those that require microsurgical skills. Table 2 does not include brachial plexus exploration and reconstruction surgeries, due in part to the fact that the hospital lacks a surgical microscope, which is required for brachial plexus reconstructions, and also due to the fact that Nicaraguan patient with brachial plexus birth palsy Brigada may present in a delayed fashion. In the US, brachial plexus surgeries are often performed around 6 months of age in the US, and the Nicaraguan patients present to hand clinic at an older age, making them less likely to benefit from brachial plexus reconstruction and more likely to require other procedures to minimize the sequelae of brachial plexus birth palsies (e.g., tendon transfers and contracture releases). Another difference is that Nicaraguan patients with syndromic congenital anomalies may be less likely to undergo surgery, as these patients may have a greater risk of anesthesia that precludes their ability to undergo surgery safely without the advanced monitoring and critical care technology available in the US. Several such patients in need of surgical intervention have been transferred to hospitals in the US where PHSG surgeons are able to care for them safely. Finally, surgeries requiring advanced technology, such as corrective forearm osteotomies using Materialise (Materialise NV, Leuven, Belgium), which utilizes 3D CT scanning, 3D printing, and advanced software technology to plan complex forearm reconstructions, are unable to be performed in Nicaragua due to the limited or absent availability of these resources.

## Discussion

### Sustainability

In addition to the mentor model of surgical education we have described here, several other methods to improve the availability and capacity of surgical providers in resource-poor environments are have been utilized, including: direct provision of surgical services (mission trips of visiting surgeons to resource-poor countries); fellowships (medical providers from resource-poor countries travel to the US to obtain experience and training not available in their home country); and attendance at international conferences and courses (which provide learning opportunities in the form of lectures and surgical simulations) (21). Each of these strategies, while beneficial, has certain limitations. Direct provision of surgical services (i.e., “parachute trips”) does not expand the skills or ability of local providers. Most US fellowships for foreign medical providers are limited to observerships, which do not allow hands-on training, and the provision of surgical services is not conducted in the context of their local resources, often relying on expensive technology that is not available in their home institution. Finally, international conferences and courses, like observerships, require substantial commitment of both time and resources, which is not feasible for many providers.

In contrast to the above surgical experiences, the training model we have implemented in Nicaragua results in sustainable delivery of health-care services to children with hand conditions. As Jairo J. Rios Roque’s responsibility and ability to independently care for patients in clinic and the operating room increase, patients with congenital and acquired hand conditions will be able to receive appropriate care from Jairo J. Rios Roque directly, throughout the year, without the services of the Brigada. Moreover, he will be able to educate and train the Nicaraguan residents and orthopedic surgeons in the care of patients with these conditions. Finally, the next step in sustainable surgical education is to establish research initiatives that investigate the outcomes of the treatment provided, to publish the results of these investigations, and allow the study findings to inform surgical decision making. Currently, we are planning to include Hospital La Mascota in the development of registry of congenital hand differences, which would ultimately be used to determine the outcomes and effective of surgical treatments for these conditions. Because Hospital La Mascota is the sole provider of surgical services for these complex conditions, a registry of the patients treated here will provide unique and comprehensive perspective into the incidence of these conditions, the need for surgical treatment, and outcomes of operative interventions; this could then be compared to similar registries in the US, enhancing our understanding of how to best improve the hands and lives of patients with these conditions.

## Conclusion

The disparity between the need for surgical care and its availability in resource-poor countries is substantial, which has far reaching consequences for individual, social, political, and economic development. Although the reasons for this disparity are complex, solutions should focus on sustainable ways to increase the supply of scarce resources. In Nicaragua, as in other developing nations, there is an unmet need for surgeons adequately trained in the management of pediatric upper extremity surgery. We believe that the model of education and training presented here is a self-sustaining solution to lack of adequately trained pediatric hand and upper extremity surgeon in Nicaragua, which can be applied to other surgical specialties in other environments. We are hopeful that the establishment of such training models improves surgical education, enables and empowers surgeons in resource-poor environments to provide of desperately needed surgical care, and improves both the health and the lives of the patients treated.

## Author Contributions

MM: primary author, responsible for data collection and analysis, drafting the work, approval of final work for submission and publication, and agreed to be accountable for all aspects of the work. JR: contributed substantially to the data collection and interpretation, made important intellectual contributions through the writing and revision processes, approved the final work for submission and publication, and agreed to be accountable for all aspects of the work. GZ: made considerable contributions to the conception and design of the training program and the written manuscript describing this program, participated in data interpretation and in critical analysis of the written work in the revising processes, approved the final work for submission and publication, and agreed to be accountable for all aspects of the work. MJ: responsible for initiation and design of the training program, conceived the idea for the written manuscript describing this program, participated in data interpretation and in critical analysis of the written work in the revising processes, approved the final work for submission and publication, and agreed to be accountable for all aspects of the work.

## Conflict of Interest Statement

The authors declare that the research was conducted in the absence of any commercial or financial relationships that could be construed as a potential conflict of interest.
